# Acute pharmacodynamic effects of pimobendan in client-owned cats with subclinical hypertrophic cardiomyopathy

**DOI:** 10.1186/s12917-021-02799-9

**Published:** 2021-02-23

**Authors:** Maureen S. Oldach, Yu Ueda, Eric S. Ontiveros, Samantha L. Fousse, Lance C. Visser, Joshua A. Stern

**Affiliations:** 1 Department of Medicine and Epidemiology, School of Veterinary Medicine, University of California-Davis, One Shields Avenue, Davis, CA 95616 USA; 2 Department of Clinical Sciences, College of Veterinary Medicine, North Carolina State University, Raleigh, NC 27606 USA

**Keywords:** Feline, Pharmacodynamics, HCM, Obstruction, Inotrope

## Abstract

**Background:**

Prior studies have suggested that pimobendan is associated with several positive effects in cats, including improved survival in cats with congestive heart failure and improved left atrial function in research colony cats with hypertrophic cardiomyopathy (HCM) and normal cats. However, there is still a paucity of pharmacodynamic data refuting or supporting the use of pimobendan in a clinical cat population. This clinical trial aimed to evaluate the pharmacodynamic effects and tolerability of a single dose of pimobendan in cats with HCM. Echocardiograms and Doppler-derived systolic blood pressures were performed in 21 client-owned cats with subclinical HCM at baseline and 90-min after oral administration of 1.25 mg of pimobendan (Vetmedin). Seven additional cats were evaluated post-placebo administration to account for intra-day variability.

**Results:**

Heart rate, systolic blood pressure, and murmur grade were not significantly different between baseline and post-pimobendan evaluations. Left auricular blood flow velocity, left atrial size, and left ventricular fractional shortening were not significantly different between baseline and post-pimobendan evaluations.

Mean (± standard deviation) tissue Doppler peak systolic velocity of the mitral annulus was significantly higher following pimobendan (7.4 cm/s ± 1.5 vs 8.5 ± 1.6; *p* = 0.02). Median (min, max) left-ventricular outflow tract maximum velocity was significantly higher following pimobendan [1.9 m/sec (1.5, 3.4) vs 2.6 m/sec (2.0, 4.0); *p* = 0.01]. Mean right-ventricular outflow tract maximum velocity was also significantly higher following pimobendan (1.5 m/s ± 0.51 vs 2.0 ± 0.53; *p* = 0.004). Mean left atrial fractional shortening was significantly higher following pimobendan (28% ± 6 vs 32% ± 7; *p* = 0.02).

No adverse events were observed following pimobendan administration.

Right ventricular outflow tract velocity was significantly higher following placebo in control cats (1.02 ± 0.21 versus 1.31 ± 0.31; *p* = 0.01). No other significant differences were detected.

**Conclusions:**

In client-owned cats with HCM, pimobendan acutely increased left atrial function and mildly increased left ventricular systolic function. Left ventricular outflow tract velocity was increased after pimobendan. Pimobendan was well tolerated in the acute setting in cats with HCM. The findings of this prospective, acute-dosing study confirm previous findings in research animals and retrospective analyses and suggest that chronic dosing studies are safe and warranted.

## Background

Pimobendan is a benzimidazole pyridazinone drug that has become part of standard therapy for dogs with congestive heart failure, pre-clinical dilated cardiomyopathy and pre-clinical degenerative valvular disease [1–6]. By sensitizing cardiac troponin C to calcium and also acting as a phosphodiesterase III inhibitor, pimobendan has both a positive inotropic effect and a balanced vasodilatory effect, improving cardiac output without any documented pro-arrhythmic effects posed by other positive inotropes such as digoxin or dobutamine [2, 7].

Numerous studies have documented the beneficial effects of pimobendan in improving quality of life, prolonging time to onset to congestive heart failure, and improving congestive heart failure control in dogs with degenerative valvular disease and dilated cardiomyopathy [1, 3, 4].

This benefit is not as well established or accepted in cats despite its frequent clinical use. Hypertrophic cardiomyopathy (HCM) is the most common cardiac disease affecting the domestic cat, with reported prevalence of approximately 15% [8, 9]. HCM is a disease that involves abnormal thickening of the ventricular myocardium (predominantly left) and concurrent myocardial fibrosis and stiffening with a nondilated left ventricular chamber [10, 11]. Cats with HCM have a preserved ejection fraction and develop diastolic dysfunction which can lead to cardiac decompensation. Although the disease course is variable, cats affected with HCM have been shown to carry a 23.8% risk of developing congestive heart failure and an 11.3% risk of developing an arterial thromboembolism [12]. Given this high disease prevalence and complication risk, therapies to mitigate risk of morbidity and mortality are needed.

In a prior retrospective case-control study, cats with HCM and CHF receiving pimobendan lived longer than those that did not receive pimobendan as part of their routine heart failure therapy [13]. This study suggested that pimobendan may be beneficial as a part of standard CHF therapy in cats. In addition, a recent placebo-controlled, blinded, crossover study assessed the acute pharmacodynamic effects of a single oral dose of pimobendan in a research colony of cats with HCM and found a significant increase in the left atrial function, as assessed by the left atrial fractional shortening (LAFS) [14]. However, this study was limited in that cats were genetically related and had to be sedated for their examinations. Another study investigating the effects of a single dose of pimobendan in healthy cats further corroborates these positive left atrial function effects by demonstrating an increase in left auricular flow velocity, LAFS, and a reduction in the minimal LA dimension [15]. Reduced LA function, as assessed by LAFS has been associated with CHF and an increased risk for thromboembolic disease and death in cats with HCM [16, 17]. Despite these findings, the recommendation for use of pimobendan in acute or chronic feline heart failure management is reported as “could be considered, provided dynamic left ventricular outflow tract obstruction is absent” with a reported low level of evidence in a recent veterinary cardiologist consensus statement on feline cardiomyopathies [11].

Another potential benefit of pimobendan could lie in its positive lusitropic effects, which have been documented in dogs and humans [18, 19]. These potential effects could be particularly important in patients with HCM given the disease pathophysiology is primarily defined by diastolic dysfunction. A prior study was unable to assess diastolic function due to the cats’ persistent tachycardia during the examinations [14].

To complicate the disease further and contribute to the controversy surrounding pimobendan use with HCM, 45.7–67% of cats with HCM presenting to referral hospitals have dynamic left ventricular outflow tract obstructions (LVOTO) [20, 21]. Dynamic LVOTO can result from either systolic anterior motion of the anterior mitral valve leaflet (SAM), asymmetric septal hypertrophy, or a mid-ventricular obstruction from hypertrophy of the mid-left ventricular myocardium or papillary muscles. Dynamic LVOTO is a major cause of symptoms associated with HCM in humans affected by the disease, including angina, exercise intolerance, and syncope [22, 23]. The role of dynamic LVOTO in feline HCM and any relationship to clinical signs of disease remains unknown. There is concern that a positive inotrope that potentially reduces cardiac afterload, such as pimobendan, could induce or exacerbate LVOTO, and therefore is another reason why continued studies are required for understanding the drug’s possible role in treating feline HCM [24]. This concern has led to controversy surrounding the use of pimobendan in feline HCM and left practitioners to interpret a plethora of largely retrospective data and expert opinion. In a recent prospective study, a single dose of oral pimobendan did not significantly induce or exacerbate LVOTO and was well tolerated [14]. This study was limited, however, because the cats were all purpose-bred and genetically similar; they were also sedated for their examinations and even so, were very sympathetically driven throughout their exams. Despite the controversy, there is a paucity of documented adverse effects described for the use of pimobendan in cats with LVOTO. In a study by Gordon and colleagues, a single cat with complex congenital disease and CHF experienced worsening of already existent systemic hypotension that was hypothesized to be secondary to pimobendan [25]. Gordon and colleagues thus recommended that caution be exercised when using this drug in cats with LVOTO and this language has been carried forward into multiple review articles and the recent feline consensus statement [11, 26]. Since the time of this original publication by Gordon and colleagues numerous retrospective studies have failed to demonstrate increased incidence of LVOTO or drug-induced hypotension [13, 27, 28]. The most recent of these studies is the largest to date, showing retrospectively that cats receiving pimobendan had no adverse hemodynamic side effects, regardless of their presence or absence of LVOTO [27]. A persistent challenge to evaluating the impact of pimobendan on the presence or absence of LVOTO is the lack of a standard definition for LVOTO in the cat and, perhaps more importantly, a clinically relevant level of induced LVOTO that should preclude the use of this therapy due to significant hemodynamic consequence. This definition was notably absent from the recent ACVIM consensus statement [11]. Therefore, it is imperative that pimobendan’s effects on cats with HCM be prospectively evaluated in a more heterogeneous, un-sedated, client-owned population to better determine the clinical relevance of these findings.

The present study aimed to investigate the acute pharmacodynamic effects of pimobendan in subclinical HCM-affected cats while mitigating some of the limitations of a prior study [14]. To do this, we aimed to use echocardiography and Doppler-derived systolic blood pressure (SBP) to assess the effects of a single oral dose of pimobendan in a heterogeneous, client-owned, non-sedated cat population with HCM to better determine the drug’s cardiac effects, tolerability, and potential clinical utility. We hypothesized that pimobendan would be well tolerated, that it would not induce or exacerbate dynamic outflow tract obstructions, that it would improve left atrial function as assessed by LAFS, and that it would improve indices of diastolic function assessed by echocardiography. This study prospectively addresses critical questions arising from multiple retrospective data sets regarding the safety and pharmacodynamic profile of pimobendan in cats with HCM.

## Results

### Treatment group

21 Client-owned cats were recruited from the patient population presenting to the University of California Veterinary Medical Teaching Hospital Cardiology service. Cats were presented for evaluation of previously diagnosed or suspected cardiac disease based on prior echocardiography, elevated NT-ProBNP, presence of a murmur, or cardiomegaly on thoracic radiographs. All cats had maximal segmental or uniform wall thickness ≥ 6 mm in the absence of systemic hypertension (SBP ≥ 180 mmHg) and hyperthyroidism. Most of the cats were domestic shorthair (*n* = 15) with a small number of domestic longhair (*n* = 3), Himalayan (n = 1), Maine coon (n = 1) and Siamese (n = 1)). Median age of the cats was 6.5 years (range 1.6 years – 15.6 years). Most cats were castrated males (*n* = 15) and the remaining cats were spayed females (*n* = 6). Median body weight was 5.8 kg (range 2.9–7.7). All cats had subclinical disease; two cats were receiving an angiotensin converting enzyme inhibitor (benazepril *n* = 1; enalapril n = 1) and clopidogrel (*n* = 2) due to previously diagnosed left atrial enlargement. Other medications received included inhaled fluticasone^1^ to treat feline asthma (*n* = 1), cyclosporine^2^ for atopic dermatitis (n = 1), and fluoxetine (n = 1). No changes to medications were made within 1 month of study enrollment.

There were systolic heart murmurs in 18/21 at baseline and 20/21 cats after pimobendan; the number of cats with murmurs was not significantly different (*p* = 0.50). Grade of the murmur was also not significantly different [baseline 3 [2, 4] versus post-pimobendan 3 [3, 4]; *p* = 0.125] (Table 1). Heart rate, SBP, left ventricular segmental wall thickness, and left atrial size as assessed by maximum left atrial dimension was not significantly different between baseline and post-pimobendan evaluations (Table 1).

**Table 1:** Descriptive Values for Pimobendan Treatment Group

	Baseline	Post-pimobendan	P value
Murmur^2^ [(median grade (IQR)]	3 (2,4)	3 (3,4)	0.13
HR^1^ (mean bpm ± SD)	192.7 ± 29.76	198.5 ± 24.09	0.29
Thickest wall segment^1^ (mean mm ± SD)	7.28 ± 0.844	6.9 ± 0.853	0.89
Systolic blood pressure^2^ [median mmHg (IQR)]	125 (109, 130)	127 (113.5, 136.5)	0.36
LAlax^1^ (mean mm ± SD)	14.7 ± 2.66	15.15 ± 2.66 1.2)	0.20
LVIDd^1^ (mean mm ± SD)	14.18 ± 1.38	14.51 ± 2.22	0.37

^1^Paired T test

^2^Wilcoxon matched-pairs signed rank test

IQR, interquartile range; HR, heart rate; bpm, beats per minute; SD, standard deviation; LA, left atrium

Categorical data are presented in Table 2. No adverse effects were observed in any of the cats. There were significantly more cats with LVOTO, defined with Vmax of > 1.9 m/s, following pimobendan (Table 2). The number of cats with level 2 LVOTO, defined with Vmax of > 2.74 m/s, was not significantly different (Table 2). There was no significant difference noted between the number of cats with SAM or mid-LVOTO at baseline versus post-pimobendan (Table 2).

**Table 2:** Categorical Variables for Pimobendan Treatment Group

	Positive before and after pimobendan (n)	Positive before and negative after pimobendan (n)	Negative before and positive after pimobendan (n)	Negative before and negative after pimobendan (n)	P value
Level 1 LVOTO^3^ (Vmax > 1.9 m/s)	10	0	7	4	0.02
Level 2 LVOTO^3^ (Vmax > 2.74 m/s)	5	0	3	13	0.25
SAM^3^	9	0	5	7	0.07
Mid-LVOTO^3^	3	1	7	10	0.08
RVOTO^3^	9	0	10	2	0.004
VPCs^3^	1	3	2	15	1.00
Murmur^3^	18	0	2	1	0.50

^3^McNemar Test

LVOTO, left ventricular outflow tract obstruction; SAM, systolic anterior motion of the mitral valve; DRVOTO, dynamic right ventricular outflow tract obstruction; VPCs, ventricular premature complexes

**Fig. 1:**
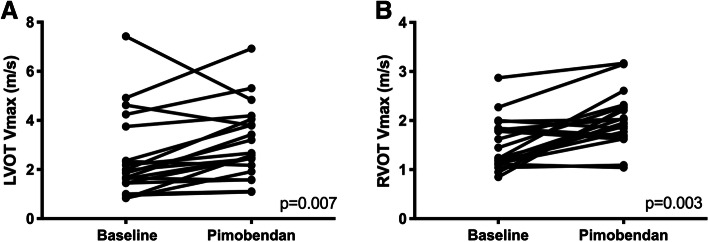
A) A Wilcoxon matched-pairs signed rank test comparison of maximal left ventricular outflow tract velocity (LVOT Vmax) (m/sec) between baseline and post-pimobendan evaluations. LVOT Vmax was significantly higher post-pimobendan (*p* = 0.007). B) A paired t-test comparison of maximal right ventricular outflow tract velocity (RVOT Vmax) (m/sec) between baseline and post-pimobendan evaluations. RVOT Vmax was significantly higher post-pimobendan (*p* = 0.003)

Functional echocardiographic variables are displayed in Table 3. LVOT Vmax was significantly higher following pimobendan [baseline 1.91 m/s (1.45, 3.4) versus post-pimobendan 2.63 m/s (1.98, 3.99) *p* = 0.007; Fig. 1a]. The heart rate at the time of LVOT Vmax was also significantly higher (baseline 190 ± 24 bpm versus 201 ± 28 bpm; *p* = 0.006). Similarly, RVOT Vmax was also increased after pimobendan (baseline 1.5 m/s ± 0.51 m/s versus following post-pimobendan 2.0 ± 0.53 m/s; *p* = 0.003, Fig. 1b). The heart rate at time of RVOT Vmax was significantly increased (baseline 185 bpm ± 25 versus post-pimobendan 197 bpm ± 27; *p* = 0.044).

**Table 3:** Functional Assessment for Pimobendan Treatment Group

	Baseline	Post-pimobendan	P value
LAFS 2-D^1^ (mean% ± SD)	28.9 ± 6.3	32 ± 7.1	0.02
LVFS M-mode^1^ (mean % ± SD)	61.6 ± 9.3	65.1 ± 8.4	0.08
Mitral TDI S’^1^ (mean m/s ± SD)	0.075 ± 0.015	0.085 ± 0.016	0.02
MAPSE^2^ [median mm (IQR)]	4.43 (4.0, 5.1)	4.5 (4.3, 4.9)	0.40
Auricular flow^1^ (mean cm/s ± SD)	78.2 ± 21.53	87 ± 21.60	0.12
TAPSE^1^ (mean mm ± SD)	8.58 ± 1.35	8.86 ± 1.08	0.30
IVRT^1^ (mean ms ± SD)	42.67 ± 9.47	40.17 ± 9.58	0.34
LVOT Vmax^2^ [median m/s (IQR)]	1.91 (1.45, 3.4)	2.63 (1.98, 3.99)	0.007
RVOT Vmax^1^ (mean m/s ± SD)	1.5 ± 0.51	2.0 ± 0.53	0.003
HR at LVOT Vmax^1^ (mean bpm ± SD)	191 ± 24	201.4 ± [28]	0.006
HR at RVOT Vmax^1^ (mean bpm ± SD)	185 ± 25	197 ± 27	0.04
HR at LAFS^1^ (mean bpm ± SD)	191.9 ± 25.7	198 ± 26.0	0.29

^1^Paired T test

^2^Wilcoxon matched-pairs signed rank test

LAFS, left atrial fractional shortening; 2-D, 2-dimensional echocardiography; SD, standard deviation; LVFS, left ventricular fractional shortening; TDI, tissue Doppler imaging; MAPSE, mitral annular plane systolic excursion; IQR interquartile range, IVRT, isovolumic relaxation time, LVOT Vmax, left ventricular outflow tract maximal velocity, RVOT Vmax, right ventricular outflow tract maximal velocity

**Fig. 2:**
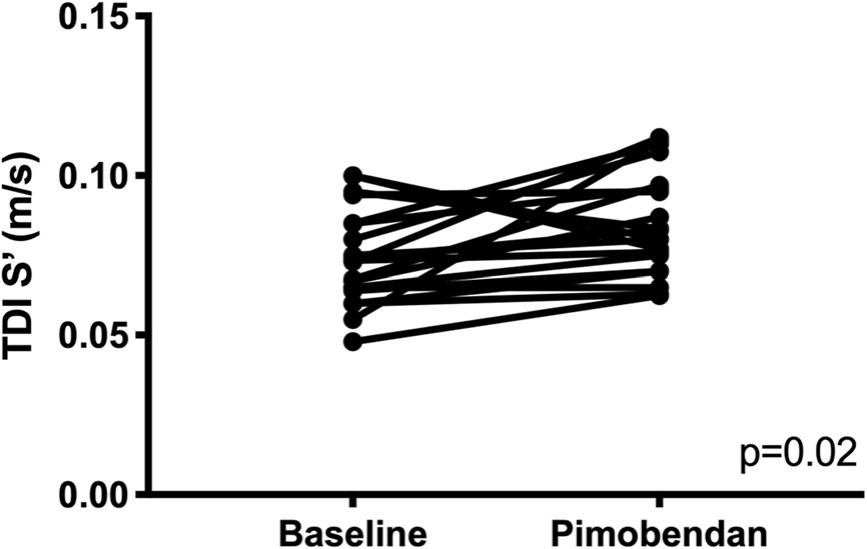
A paired t-test comparison of tissue Doppler imaging of the systolic peak (TDI S′) (m/sec) of the lateral mitral annulus between baseline and post-pimobendan evaluations. TDI S′ was significantly higher post-pimobendan (*p* = 0.020)

Mitral PW TDI S′ was significantly higher following pimobendan (baseline 0.075 m/s ± 0.015 versus post-pimobendan 0.085 m/s ± 0.016; *p* = 0.02) (Fig. 2). LV fractional shortening (LVFS) was not significantly different. Tricuspid annular plane systolic excursion (TAPSE) and mitral annular plane systolic excursion (MAPSE) showed no significant difference following pimobendan.

**Fig. 3:**
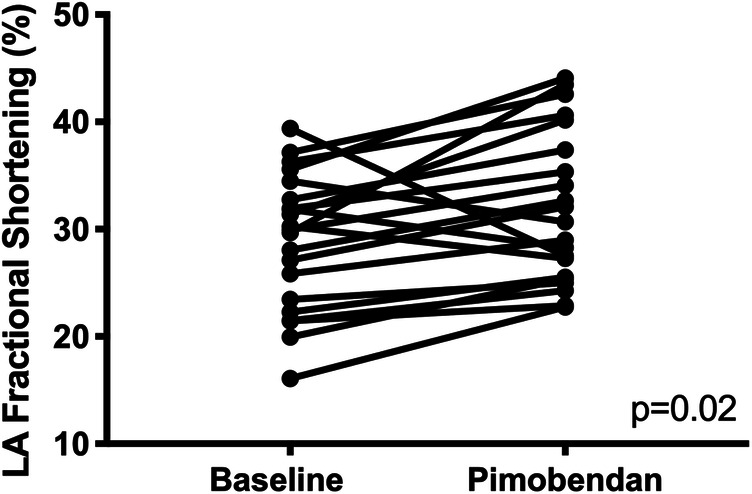
A paired t-test comparison of left atrial (LA) fractional shortening (%) between baseline and post-pimobendan evaluations, as assessed by 2-dimensional echocardiography. LA fractional shortening was significantly higher post-pimobendan (p = 0.02)

LA fractional shortening (LAFS) was significantly increased following pimobendan (baseline 28.9% ± 6.3 versus post-pimobendan 32.0% ± 7.1; p = 0.02) (Fig. 3). Heart rate at the time of LAFS measurement was not significantly different (baseline 192 ± 26 bpm versus 198 ± 26 bpm; *p* = 0.29). Auricular flow velocity was not significantly different following pimobendan.

IVRT was not significantly different before or after pimobendan (Table 3). Other diastolic function assessment, such as PW TDI E’ and A’ assessment and transmitral spectral Doppler studies were attempted but due to tachycardia and insufficient numbers of cats with separation of their E/E’ and A/A’ waves, statistical assessment of these values could not be performed.

### Control group

Seven client-owned cats with subclinical HCM based on the aforementioned criteria were enrolled as controls. Median age was 3.5 years with a range of 1.6–13.7 years. Most (*n* = 6) were domestic shorthair cats with one domestic longhair cat. Most cats were castrated males (n = 6) with one spayed female. Median body weight was 5.5 kg (range 3.9–6 kg). No cats were receiving any medications and none had clinically apparent systemic disease. Segmental wall thickness at baseline was 6.42 mm ± 0.57, which did not significantly differ from post-placebo (6.61 ± 0.59).

2/7 cats had LVOTO at their baseline exam, and 4/7 had an LVOTO after receiving placebo; this difference was not statistically significant (*p* = 0.48). One cat had a level 2 LVOTO (LVOT Vmax > 2.7 m/s), which was present at baseline (LVOT Vmax 3.12 m/s) and post-placebo (LVOT Vmax 3.19 m/s). No control cat had an RVOTO at baseline and 1/7 developed RVOTO following placebo; this was not significantly different compared with baseline (*p* = 1.00).

**Fig. 4:**
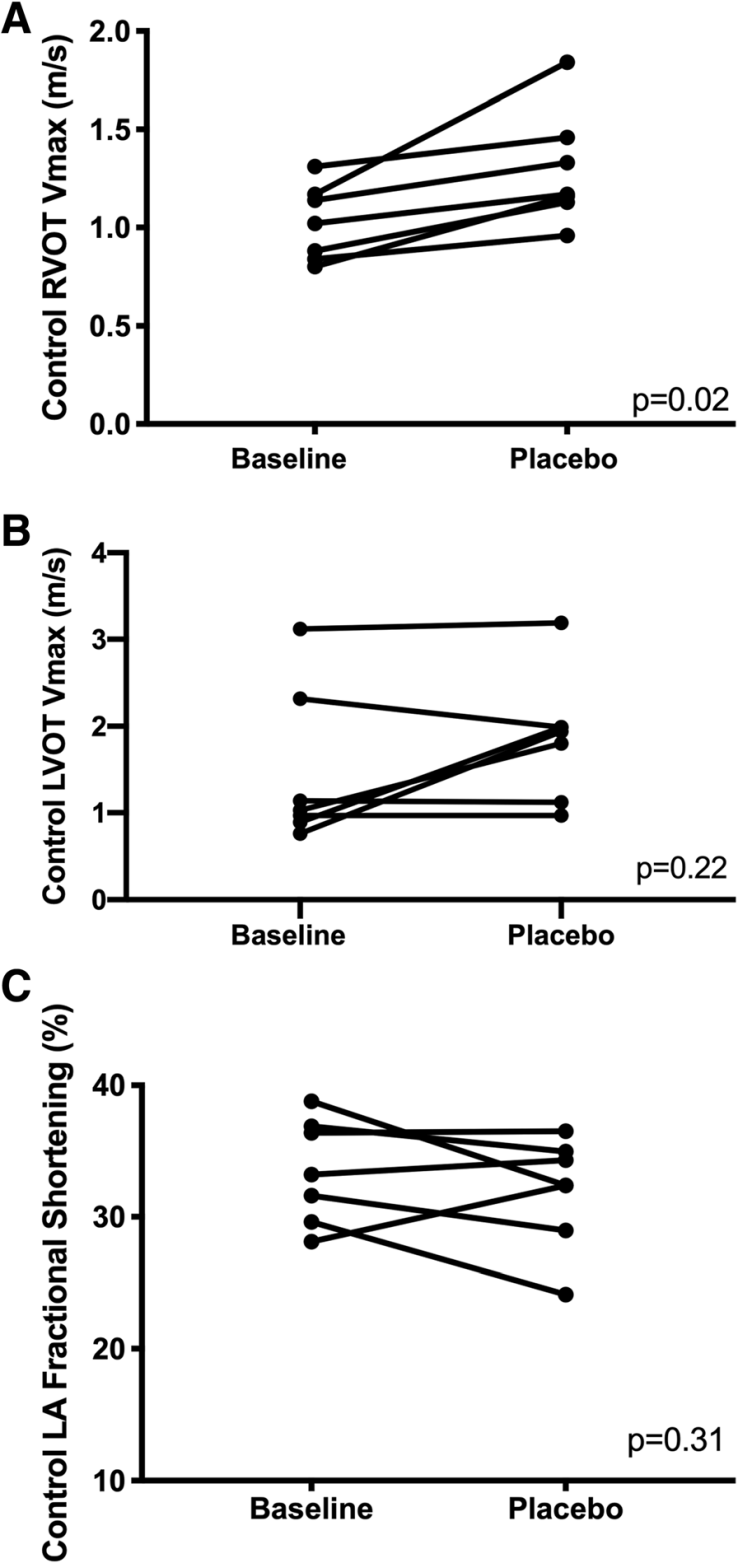
A) A paired t-test comparison of maximal right ventricular outflow tract velocity (RVOT Vmax) (m/sec) between baseline and post-placebo evaluations of the control cats. RVOT Vmax was significantly higher post-placebo (p = 0.02) B) A Wilcoxon matched-pairs signed rank test comparison of maximal left ventricular outflow tract velocity (LVOT Vmax) (m/sec) between baseline and post-placebo evaluations of the control cats. No significant difference was identified (*p* = 0.22). C) A paired t-test comparison of left atrial (LA) fractional shortening (%) between baseline and post-placebo evaluations, as assessed with 2-dimensional echocardiography. No significant difference was identified (*p* = 0.06)

The functional echo data for control cats are presented in Table 4. RVOT Vmax was significantly increased following placebo (baseline 1.02 m/s ± 0.19 versus post-placebo 1.29 ± 0.29; *p* = 0.01) (Fig. 4a). LVOT Vmax was not significantly increased following placebo [baseline 1.03 m/s (0.89, 2.32) versus post-placebo 1.94 m/s (1.12, 1.99); *p* = 0.22] (Fig. 4b). HR at RVOT and LVOT Vmax was not significantly different following placebo (Table 4). LAFS also showed no significant difference at baseline compared with placebo (Table 4) (Fig. 4c). There were also no significant differences in LVFS and mitral TDI S′ between baseline and post-placebo (Table 4).

**Table 4:** Functional data for Placebo-control Group

	Baseline	Post-placebo	P value
LAFS 2-D^1^ (mean% ± SD)	33.51 ± 4.00	31.95 ± 4.2	0.31
Mitral TDI S’^1^ (mean m/s ± SD)	0.07810 ± 0.0149	0.0767 ± 0.0234	0.83
LVOT Vmax^2^ [median m/s (IQR)]	1.03 (0.89, 2.32)	1.94 (1.12, 1.99)	0.22
RVOT Vmax^1^ (mean m/s ± SD)	1.02 ± 0.19	1.29 ± 0.29	0.01
HR at LVOT Vmax^1^ (mean bpm ± SD)	201.7 ± 20.55	209.3 ± 23.44	0.48
HR at RVOT Vmax^1^ (mean bpm ± SD)	187.3 ± 10.86	201.7 ± 25.64	0.10

^1^Paired T test

^2^Wilcoxon matched-pairs signed rank test

LAFS, left atrial fractional shortening; 2-D, 2-dimensional echocardiography; SD, standard deviation; LVFS, left ventricular fractional shortening; TDI, tissue Doppler imaging; LVOT Vmax, left ventricular outflow tract maximal velocity, IQR interquartile range, RVOT Vmax, right ventricular outflow tract maximal velocity

### Pimobendan treatment group versus control group

Percent change following pimobendan treatment and placebo treatment groups for those values that showed significant changes following pimobendan are included in Table 5. Percent change of left atrial fractional shortening from baseline versus post-pimobendan and baseline versus post-placebo was significantly different [pimobendan treatment group percent change 12.08% (6.25, 17.43) versus control group − 5.55% (− 19.75, 3.27); *p* = 0.03]. There were no significant differences in percent change of mitral TDI S′, LVOT Vmax, RVOT Vmax, HR at LVOT Vmax, or HR at RVOT Vmax between treatment and control groups.

**Table 5:** Percent Change in Functional Measures in Pimobendan versus Placebo Groups

	Post-pimobendan	Post-placebo	P value
LAFS 2-D^4^ [median % change (IQR)]	12.08 (6.25, 17.43)	−5.77 (− 19.75, 3.28)	0.03
Mitral TDI S^5^ (mean % change ± SD)	10.76 ± 19.85	−6.42 ± 23.07	0.11
LVOT Vmax^5^ (mean % change ± SD)	20.08 ± 28.92	20.39 ± 31.51	0.96
RVOT Vmax^5^ (mean % change ± SD)	22.6 ± 23.8	19.92 ± 10.25	0.68
HR at LVOT Vmax^5^ (mean % change ± SD)	4.67 ± 6.7	2.8 ± 12.8	0.72
HR at RVOT Vmax^5^ (mean bpm ± SD)	5.1 ± 12.9	6.26 ± 9.30	0.80

^4^Mann-Whitney U Test

^5^Welch’s T Test

LAFS, left atrial fractional shortening; 2-D, 2-dimensional echocardiography; SD, standard deviation; TDI, tissue Doppler imaging; LVOT Vmax, left ventricular outflow tract maximal velocity, IQR interquartile range, RVOT Vmax, right ventricular outflow tract maximal velocity

## Discussion

This study corroborates prior evidence that pimobendan improves left atrial function, as assessed by LAFS. Two prior studies also had this finding: one study showed an improved LAFS in research-colony of cats with HCM following a single dose of pimobendan, and another study demonstrated improved LAFS in healthy cats following a single pimobendan dose [14, 15]. This repeatable finding has now been observed in an un-sedated, heterogeneous, clinical population of cats with naturally occurring HCM. Given this change was identified during comparison of baseline and post-pimobendan exams as well as during comparison of percent changes in LAFS in the post-pimobendan treatment group and post-placebo treatment group, this likely represents a true drug effect and not a chance effect or effect of time. This improvement in LA function may represent a contributor to the previously identified survival benefit for cats that received pimobendan as a part of their heart failure therapy [13]. LAFS has been shown to be reduced in congestive heart failure, and has been shown to be correlated with an increased risk for thromboembolic disease and death, so improving left atrial function could mitigate some of this risk and improve prognosis [16, 17]. This effect could represent a direct effect on the atrial myocardium or could be an indirect reflection of improved left ventricular diastolic function. Future studies aiming to evaluate if the effect on LAFS is of clinical relevance are warranted.

Comprehensive diastolic function assessment was severely limited in this study due to persistent tachycardia and low numbers of cats that had separation of E and A waves on mitral annular TDI and transmitral flow. IVRT was the only direct diastolic measure that was consistently acquired and was not significantly different following pimobendan administration. This is consistent with findings from prior studies [14, 15]. Persistent tachycardia also limited diastolic function assessment in the prior study assessing pharmacodynamics of pimobendan in HCM cats [14]. Improved diastolic function following pimobendan has been shown in dogs with tachycardia-induced cardiomyopathy and in people with myocardial failure and warrants further research in cats, as enhanced lusitropy would be desirable in feline HCM [18, 19]. Additionally, given that feline HCM is an excellent translational model for human HCM, further assessment of pimobendan’s potential lusitropic effects could have implications for human HCM therapy, as inodilators have not been investigated in humans with HCM [29]. To evaluate for a possible diastolic effect, pharmacologic slowing of the heart rate will likely be necessary.

Left auricular flow velocity was not significantly increased by pimobendan in this study, which is congruent with the prior study evaluating pimobendan in HCM cats, but not consistent with findings from the study in normal cats [14, 15]. This may represent physiologic differences related to the HCM disease pathology; alternatively, the increased auricular flow velocity noted in the 2020 Toaldo et al. study could have been related to heart rate, which was significantly increased in the study following pimobendan, whereas the heart rate at baseline and at the time of LAFS measurement was not significantly different in this study, which is similar to the 2019 Oldach et al. study that also did not show a heart rate effect [14, 15]. Given that this study and the prior study with HCM affected cats showed increases in LAFS but not auricular flow velocity, it is possible that pimobendan increases the extent of left atrial contraction but not the rate of contraction in HCM affected cats [14]. It is also possible that this study and the prior studies have been underpowered to detect a change in left auricular flow velocity.

Pimobendan was associated with a mild increase in only one systolic function measure, the mitral annular TDI S′; however, this change may be an effect of time and not a true drug effect, given that the percent change in TDI S′ was not significantly different between pimobendan treatment group and the control group. LVFS was not significantly increased following pimobendan in this study; this is consistent with findings from prior studies in HCM-affected cats and healthy cats, which failed to show significant changes in LVFS at any single time point [14, 15, 30]. This lack of observed systolic effect could be related to underpowering of the study or could be related to the physiologic mechanism of HCM, which involves increases in sarcomeric contractility with both thin and thick-filament mutation etiologies of HCM. This effect of the disease etiology may overshadow any positive inotropic effect by pimobendan at the level of the LV myocardium [31]. Thus, the inotropic effects of pimobendan on the left ventricular myocardium are likely of minimal clinical significance in this disease in the preclinical stage. It is possible that appreciable systolic function effects may be observed in cats with later-stage HCM, particularly in those that have developed reduced cardiac function, a negative prognostic factor for the disease; a study with this type of population may have yielded different results [32].

The results of this study detected a significant increase in LVOTO following pimobendan when using a cut-off value for LVOT Vmax of 1.9 m/s and did increase LVOT Vmax. This definition of LVOTO represents the upper end of normal LVOT velocity based on prior studies in normal cats and was used as a LVOTO definition in a prior study [14, 33]. Clinically relevant obstruction has not been defined in cats; however, extensive work published in the human literature has defined a left ventricular pressure gradient of > 30 mmHg as a significant obstruction, with LVOT pressure gradients higher than this putting patients at increased risk for congestive heart failure, progression of New York Heart Association heart failure class, and cardiac-related death when compared with non-obstructed patients [34]. In the absence of an accepted definition for a clinically relevant level of obstruction in cats, the authors adopted a second level of obstruction for testing pimobendan’s effects in line with the human literature. When using > 30 mmHg pressure gradient (level 2 LVOTO) as a definition of obstruction, equating to an LVOT Vmax of > 2.74 m/s, our study did not detect a significant increase in level 2 LVOTO following pimobendan. It is important to note that the authors evaluated multiple levels of LVOTO based upon the veterinary and human literature and that these levels of obstruction were defined after this study was completed. Future studies evaluating pimobendan and LVOTO should aim to investigate a clinically relevant level of obstruction defined prior to the start of the study.

One confounding variable that should be considered in light of our findings on LVOT Vmax is an increase in heart rate at the time of LVOT Vmax measurement, which was significantly higher following pimobendan. Although heart rates acquired during the first half of the echo (baseline HR) were not significantly different following placebo, the LVOT Vmax was recorded during the second half of the echocardiogram, when sympathetic tone was likely higher due to the stress of prolonged handling; this may be heightened in the second examination of the day. Thus, the elevation in LVOT Vmax and LVOTO may be related to increased sympathetic tone and elevated heart rate, and not solely due to pimobendan’s drug effect. This concept is further supported by the comparison of percent change in heart rate and LVOT Vmax between the pimobendan treatment group and control group. Given the percent change in baseline HR, LVOT Vmax, and HR at LVOT Vmax were not different between groups, the factor of time (second echo of the day) is likely a contributor to the findings of increased LVOT Vmax and LVOTO. However, it is important to note that our control population was smaller than the treatment population, which may have introduced type II error. Thus, the absence of significant differences between treatment groups should not be interpreted as absence of drug effect.

Similarly, pimobendan treatment was also associated with a significant increase in RVOTOs and RVOT Vmax. However, an increase in RVOT Vmax was also noted in the control group, and the percent change of RVOT Vmax was not significantly different between post-pimobendan and post-placebo evaluations. Given this, and given that percent change in heart rate was also not significantly different between post-pimobendan and post-placebo evaluations, an effect from elevated sympathetic tone as a contributor to these changes is likely. This is further supported by lack of a significant difference in RVOTO/RVOT Vmax in the previous placebo-controlled crossover study of pimobendan in HCM cats [14]. However, given our small sample size and disparate sample sizes between treatment groups, it is not possible to completely isolate drug effect from the effect of time on RVOT velocity.

Pimobendan treatment was not associated with any significant change in SBP in this study. A prior study assessing pimobendan in healthy cats showed a significant reduction in non-invasive diastolic and mean blood pressure as assessed by oscillometry [30]. However, oscillometric blood pressure devices have shown poor correlation to direct arterial blood pressure assessment in cats, with wide variability between devices [35, 36]. Doppler-derived systolic blood pressure has been shown to meet veterinary standards for correlation with invasive systolic blood pressure, so results in this study are likely to approximate true systemic blood pressure [37]. However, further research using invasive blood pressure assessment may be considered to better characterize pimobendan’s blood pressure effects in cats with HCM.

Based on our results, a single dose of pimobendan appears to be safe in cats with hypertrophic cardiomyopathy. No cats in this study experienced an identifiable adverse reaction, regardless of obstruction status. There was no significant difference in the presence of arrhythmias before or after pimobendan. Additionally, no cat became hypotensive following pimobendan, which is a theoretical concern for administering the drug to cats with LVOTO. The lowest SBP observed following pimobendan was 107 mmHg, which was actually a slight increase from that cat’s baseline (99 mmHg). Therefore, there is no evidence from this study to suggest that administering a single dose of pimobendan in cats with hypertrophic cardiomyopathy is contraindicated. However, given there were a relatively small number of cats with level 2 LVOTO, future studies with a higher number of more severely obstructed cats with repeated dosing is indicated to better determine safety of pimobendan in cats with HCM.

This study has several limitations to address. To start, drug levels were not measured in this study, so we were unable to correlate plasma concentrations to physiologic effect. Additionally, we chose to use a single dose (1.25 mg) of pimobendan for all cats, regardless of size. Although this is a commonly used clinical dose of the drug [11], the wide range of body weights and variable mg/kg dosing may have led to disparate effects based on patient dose.

Another limitation of this study is that the treatment group corresponded with a later time point and the second echocardiogram of the day, which may have confounded results by making pimobendan drug effects indistinguishable from effects related to increased sympathetic activity from handling stress. A small group of cats administered a placebo was included to account for the effect that time spent in the hospital and receipt of an oral medication could have had on measurements, but the disparate sample sizes between pimobendan treatment group and control group limited the ability to perform powerful between-group comparisons, or even identify important changes within the control group. A double-blinded, crossover design would have circumvented these pitfalls and would have been a stronger study design. In addition, although we attempted to limit comparisons of multiple similar measures, the use of multiple comparisons could have yielded false positive results; in this study, due to small sample sizes, we did not correct for multiple comparisons due to concern for increasing likelihood of type II error. We were also limited by small sample size and by the disparately sized groups (treatment versus control). Although it is common in the veterinary literature to use smaller control populations in single-dose pharmacodynamics studies, this disparity could have introduced type I error into our results particularly in the control group [38, 39]. Therefore, lack of findings in this study cannot be assumed to represent an absence of drug effect, and further research is warranted.

Additionally, technical difficulties in measuring and performing echocardiograms on cats with asymmetrically hypertrophied ventricles make precise, accurate, and repeatable echocardiographic assessment challenging, and this could be precluding identification of subtle but potentially significant changes in cardiac function that could better elucidate pimobendan’s effects in cats. High heart rates also prevent a complete diastolic study from being assessed, which is particularly pertinent to cats with HCM, given that it is primarily a disease of diastolic dysfunction; therefore, diastolic effects of pimobendan remain unknown. Finally, the population of cats in this study is not the target population for this drug. At this point there is no evidence pimobendan is of benefit in cats prior to the onset of congestive heart failure. Therefore, a study involving only cats in congestive heart failure would be ideal, as these cats may responded differently; this may be challenging due to the fact that many cats with CHF will require initiation or changes to their therapy when evaluated, and the hemodynamic effects of CHF therapies could be a major confounder for a robust pharmacodynamic study of pimobendan.

## Conclusions

In conclusion, this study provides evidence that a single oral dose of pimobendan in cats with subclinical HCM improves LA function as assessed by LAFS and is well tolerated in the acute setting, with no cats experiencing an adverse effect. Pimobendan was associated with increases in LVOT and RVOT Vmax and LVOTO defined as LVOT Vmax > 1.9 m/s. However, this study did not show pimobendan was associated with a significant increase in level 2 LVOTO, defined as LVOT Vmax > 2.74 m/s. The cut-off value for clinically relevant LVOTO in cats with HCM is unknown and readers should interpret the increased LVOT velocity after pimobendan in light of their own clinical practice and experience. Ultimately, pimobendan’s effect on echocardiographic systolic function indices in cats with HCM appears to be mild. This prospective study confirms that a future chronic-administration placebo-controlled clinical trial is indicated to better characterize pimobendan’s effects and tolerability and to determine its indications for clinical use in cats with HCM.

## Materials and methods

### Animals

All animal procedures were in accordance with the national Research Council Guide for the Care and Use of Laboratory Animals using protocols approved by the Institutional Animal Care and Use Committee at the University of California, Davis (protocol #20710). The required sample size for this clinical trial was estimated prior to enrollment as 20 cats. This was based on a previously reported standard deviation and expected change in the target variable of left atrial fractional shortening [14], this study’s planned primary outcome variable. Using commercially available software,^3^ sample size was calculated for a paired t-test design and a sample size of 20 pairs was determined to have a 95% power to detect a difference between means of 5.0 with a two-tailed significance level of 0.05. Thus, the treatment group for this study consisted of 21 client-owned cats with HCM who were prospectively recruited from the patient population presenting to the UC Davis Veterinary Medical Teaching Hospital for evaluation of previously diagnosed or suspected cardiac disease. Seven client-owned cats subjected to the same enrollment criteria as the treatment cats were recruited as controls. All evaluations took place in the UC Davis Veterinary Medical Teaching Hospital clinical cardiology treatment area.

### Enrollment criteria

Cats diagnosed with HCM without a history of cardiac complications of congestive heart failure, arterial thromboembolism, and/or or clinically significant arrhythmias were eligible for enrollment. Cats were identified as affected by HCM if there was segmental or diffuse left ventricular posterior wall (LVPW) or interventricular septal (IVS) end-diastolic wall thickness ≥ 6.0 mm on two echocardiographic examinations separated by 7–14 day. Absence of hypertension (SBP) > 180 mmHg, hyperthyroidism (defined as total serum thyroxine concentration of > 3.3 μg/dL, which was measured in all cats > 6 years of age) and congenital heart defects was also required. Only cats amenable to echocardiographic examination without sedation were enrolled.

Cats were excluded if they were receiving medications that had a known direct effect on myocardial function, such as anti-arrhythmic medications, theophylline, terbutaline, or pimobendan, if they had modifications to any other therapies within the prior seven days, if they received injectable steroids within the past 6 months or oral steroids within the past 2 weeks, if they were hypotensive (SBP < 100 mmHg) or if they received parenteral fluid therapy within the last 7 days. Cats with other clinically apparent systemic disease, hemodynamically significant arrhythmias, or congenital heart defects were also excluded.

Thus, cats amenable to non-sedated echocardiograms who were diagnosed with primary HCM and who were not receiving any of the therapies/changes to therapies mentioned above were included in the study.

### Experimental design

The study was prospective, where cats had both a baseline evaluation that served as an internal control and a single acute intervention of 1.25 mg pimobendan PO with a post-drug evaluation to follow.

Baseline evaluation consisted of a cardiovascular examination, SBP, and complete echocardiographic analysis. Following evaluation, 1.25 mg pimobendan^4^ was administered PO once. The 1.25 mg dose was chosen as this was the most commonly reported dose in a retrospective case series evaluating pimobendan in cats with CHF and was the dose used in two prior studies evaluating pharmacodynamics of pimobendan in cats [13–15]. Cats were not explicitly fasted, but were in hospital and not fed for a minimum of 2 h prior to receiving pimobendan. Following pimobendan administration, cats were monitored closely for adverse reactions. An adverse reaction was defined as any adverse event occurring in temporal proximity to administration of the study drug, including collapse, vomiting, diarrhea, agitation, ptyalism, arrhythmias (which were monitored for during the echocardiogram by a lead II electrocardiographic rhythm strip), and hypotension. 90 min after administration, the evaluation, including cardiovascular examination, SBP, and echocardiogram were repeated. 90 min was chosen based on prior pharmacokinetic and pharmacodynamic data [30].

All echocardiographic images were stored for offline analysis. All echocardiographic measurements were performed by a single investigator (MSO) who was blinded to the patient, time of study, and treatment versus control group at the time of analysis. Measurements and analysis were performed using Syngo Dynamics proprietary software.^5^ Due to the study design, with two sequential examinations occurring in a single day, blinding was not feasible during the physical examination and SBP.

### Controls

Seven of the cats diagnosed with HCM based on the aforementioned inclusion criteria were enrolled to account for intraday variation in these echo measurements due to hospitalization, handling, and oral medication administration. They were enrolled following enrollment of the twenty-one cats who participated in the pimobendan treatment protocol. These cats received a baseline cardiovascular examination SBP, and complete echocardiographic analysis. They then received a lactose powder filled gel capsule as a placebo. Their cardiovascular examination, SBP, and echocardiogram were completed 90 min following capsule administration.

### Echocardiographic analysis

All echocardiographic examinations were performed without sedation by a single investigator (MSO) on a Phillips EPIQ 7C ultrasound machine using a 12 MHz transducer with harmonics.^6^ Cats were gently restrained in lateral recumbency and standard imaging planes were acquired from right and left parasternal imaging windows as previously described [40]. A concurrent lead II continuous ECG was acquired and monitored for arrhythmias.

Assessment of left ventricular wall thickness was made from 2D images in the right parasternal long or short axis view using the inner edge to inner edge measurement technique. Maximal right ventricular outflow tract velocity (RVOT Vmax) was acquired from the right parasternal short axis view at the level of the heart base using continuous wave (CW) Doppler. Right ventricular outflow tract obstruction (RVOTO) was defined as color flow Doppler turbulence within proximal infundibular region of the RVOT and an RVOT Vmax of > 1.6 m/s in the absence of other cardiac pathology (such as cardiac shunts or pulmonic stenosis) as measured with CW Doppler. LVOTO was identified from a left parasternal 5-chamber view and was defined as the presence of color Doppler flow turbulence in the LVOT and a late-peaking CW Doppler profile with a velocity > 1.9 m/s. Measures of > 1.6 m/s and > 1.9 m/s were chosen to define RVOTO and LVOTO respectively as these velocities represent the upper end of normal based on a prior study of 100 healthy cats and were consistent with previous methodology reported [14, 33]. A second level of obstruction was evaluated, termed level 2 LVOTO, and was defined as LVOT velocity > 2.74 m/s, equating to a pressure gradient of 30 mmHg. This level 2 LVOTO was chosen to document effect size and was based on the human literature. An LVOT pressure gradient of greater than 30 mmHg has been well established as a threshold for clinically relevant obstruction in people, having been associated with increased risk of cardiac symptoms, progression of New York Heart Association heart failure class, and cardiac-related death [34]. Left ventricular outflow tract obstructions were differentiated into those resulting from a mid-left ventricular obstruction and those resulting from systolic anterior motion of the anterior mitral leaflet (SAM). Mid-LVOTO was identified when there was mid-ventricular hypertrophy with color-flow Doppler aliasing arising in the mid-LV during systole in combination with the spectral Doppler criteria above. The presence of SAM was evaluated on 2-dimensional (2D) assessment of the mitral valve and LVOT in the right parasternal long-axis five chamber view, and was identified when the anterior leaflet moved towards the septum and into the LVOT in systole, resulting in color-flow Doppler turbulence within the LVOT.

Additional echo measurements were acquired including the following: left atrial fractional shortening (LAFS) using a 2D assessment from the right parasternal short axis basilar view; maximal left atrial dimension (LA LAX) using a 2D assessment from the right parasternal long axis 4-chamber view in the end-systolic frame, just prior to the mitral valve opening; LV internal dimensions in systole (LVIDs) and diastole (LVIDd), which was used to calculate LV fractional shortening (LVFS); pulsed-wave Doppler imaging (PW TDI) of the lateral mitral annulus from the left apical 4-chamber view; m-mode mitral annular plane systolic excursion (MAPSE) of the septal annulus from the left apical 4-chamber view; LV isovolumic relaxation time (IVRT) using spectral PW Doppler from the left parasternal apical 5-chamber view; m-mode tricuspid annular plane systolic excursion (TAPSE) from the left parasternal apical 4-chamber view, optimized for the RV; spectral PW Doppler auricular flow velocity from the left parasternal cranial long axis view optimized for the left auricle. Transmitral spectral Doppler and diastolic tissue Doppler imaging was recorded from the left parasternal apical 4-chamber view. Transmitral spectral Doppler and diastolic tissue Doppler is frequently fused with tachycardia and when fused, these measures were not available for analysis.

Primary measures were those of left atrial function (LAFS, auricular flow velocity) and LVOT velocity.

All echocardiographic measures represent the mean of 3–5 measurement values. 5 measures were used when possible but only high-quality images were used, which limited some measures to 3 values. Baseline heart rate assessment was performed by averaging 3 R-R intervals on the rhythm ECG recorded on the m-mode images that were used for assessment of LVFS. Instantaneous heart rate was also assessed at the time of RVOT Vmax and LVOT Vmax. HR at the time of LAFS was taken as an average of 3 R-R intervals on the ECG in the cine loop used to measure LAFS.

### Statistics

Statistical analysis was performed using GraphPad Prism version 7.0 and GraphPad Quickcalcs.^7^ All variables were first analyzed for summary and descriptive statistics and tested for normality by visual inspection and D’Agostino Pearson Omnibus Normality Test for cats that received pimobendan and a Shapiro-Wilk normality test for control cats that received placebo (due to small sample size). Normally distributed data are reported as mean +/− standard deviation (SD). Non-parametric data are reported as median and interquartile range (IQR). Baseline versus post-pimobendan treatment groups were compared using a paired t test (when normally distributed) and a Wilcoxon signed-rank matched pair tests (when non-parametric). Paired categorical data were compared between baseline and post-pimobendan with a McNemar test using GraphPad Quickcalcs^7^. A *P* value of < 0.05 was considered significant.

For those measures that showed significance between baseline and post-pimobendan evaluations, percent change was calculated in these measures for both control and treatment cats. These percent changes were compared between treatment and control cats with a Welch’s t-test for parametric data and a Mann-Whitney U test for nonparametric data.

## Data Availability

The datasets used during the current study are available from the corresponding author upon reasonable request.

## Abbreviations

HCM: Hypertrophic cardiomyopathy
LA: Left atrium
LV: Left ventricle
LVOT: Left ventricular outflow tract
LVOTO: Left ventricular outflow tract obstruction
LVIDs: Left ventricular internal dimension in systole
LVIDd: Left ventricular internal dimension in diastole
RVOT: Right ventricular outflow tract
RVOTO: Right ventricular outflow tract obstruction
LAFS: Left atrial fractional shortening
2D: Two dimensional echocardiography
M-mode: Motion mode echocardiography
PW: Pulsed wave
TDI: Tissue doppler imaging
Vmax: Maximal velocity
MAPSE: Mitral annular plane systolic excursion

## Footnotes

1 Flovent, GlaxoSmithKline plc., Brentford, UK

2 Atopica, Elanco Animal Health, Greenfield, IN, USA

3 Graphpad Statmate 2.0, Graphpad, La Jolla, CA, USA

4 Vetmedin, Boehringer Ingelheim Vetmedica, Inc., St. Joseph, MO, USA

5 Syngo Dynamics Software, Siemans Medical Solutions, Malvern, PA, USA

6 Phillips Healthcare, Rancho Cordova, CA, USA

7 GraphPad Prism Software Inc., La Jolla, CA, USA
